# In Vitro Human Umbilical Vein Endothelial Cells Response to Ionic Dissolution Products from Lithium-Containing 45S5 Bioactive Glass

**DOI:** 10.3390/ma10070740

**Published:** 2017-07-03

**Authors:** Luis A. Haro Durand, Gabriela E. Vargas, Rosa Vera-Mesones, Alberto Baldi, María P. Zago, María A. Fanovich, Aldo R. Boccaccini, Alejandro Gorustovich

**Affiliations:** 1 Department of Pathology and Molecular Pharmacology, IByME-CONICET, C1428ADN Buenos Aires, Argentina; harodurand.luis@gmail.com (L.A.H.D.); bertobaldi@gmail.com (A.B.); 2 Department of Developmental Biology, National University of Salta, A4408FVY Salta, Argentina; bio_gabriela@yahoo.com.ar (G.E.V.); rosa_veramesones@yahoo.com.ar (R.V.-M.); 3 Institute of Experimental Pathology, IPE-CONICET, A4408FVY Salta, Argentina; paola_zago@yahoo.com.ar; 4 Research Institute for Materials Science and Technology, INTEMA-CONICET, B7608FDQ Mar del Plata, Argentina; mafanovi@fi.mdp.edu.ar; 5 Department of Materials Science and Engineering, Institute of Biomaterials, University of Erlangen-Nuremberg, 91058 Erlangen, Germany; aldo.boccaccini@ww.uni-erlangen.de; 6 Interdisciplinary Materials Group-IESIING-UCASAL, INTECIN UBA-CONICET, A4400EDD Salta, Argentina

**Keywords:** endothelial cell, lithium, bioactive glass

## Abstract

Since lithium (Li^+^) plays roles in angiogenesis, the localized and controlled release of Li^+^ ions from bioactive glasses (BGs) represents a promising alternative therapy for the regeneration and repair of tissues with a high degree of vascularization. Here, microparticles from a base 45S5 BG composition containing (wt %) 45% SiO_2_, 24.5% Na_2_O, 24.5% CaO, and 6% P_2_O_5_, in which Na_2_O was partially substituted by 5% Li_2_O (45S5.5Li), were obtained. The results demonstrate that human umbilical vein endothelial cells (HUVECs) have greater migratory and proliferative response and ability to form tubules in vitro after stimulation with the ionic dissolution products (IDPs) of the 45S5.5Li BG. The results also show the activation of the canonical Wnt/β-catenin pathway and the increase in expression of proangiogenic cytokines insulin like growth factor 1 (IGF1) and transforming growth factor beta (TGFβ). We conclude that the IDPs of 45S5.5Li BG would act as useful inorganic agents to improve tissue repair and regeneration, ultimately stimulating HUVECs behavior in the absence of exogenous growth factors.

## 1. Introduction

Angiogenesis is a complex morphogenetic process characterized by several cellular and molecular events that lead to the generation of new vessels from a pre-existing vascular bed [1,2,3,4]. A growing number of studies have shown that angiogenesis may be stimulated by bioactive glasses (BGs) with different chemical compositions [5,6,7,8,9,10,11,12]. Experimental evidence both in vitro and in vivo has shown the proangiogenic effects of certain ions such as Si, B, Cu, Co, and Sr released from BGs [5,6,7,8,9,10,11,12]. The incorporation of other ions with proangiogenic potential such as lithium (Li) in BGs is therefore a useful strategy to develop novel materials for regenerative medicine of vascularized tissues. 

Li is an alkaline metal with an electronic configuration of 1s^2^2s^1^, which is distributed in variable quantities in rocks, soil, and water [13,14]. Since its outer electron detaches easily, it becomes a monovalent cation (Li^+^), which is more stable and reactive, and is thus involved in different biological processes [13,14]. Li^+^ has been widely used as a drug for the prevention and treatment of neurodegenerative diseases and different psychiatric disorders such as bipolar disorder, unipolar depression, schizophrenia, and mania, because it acts on the regulation of neurotransmitters and mitochondrial function, attenuating the expression of genes associated with the signaling pathways of protein kinases A and C (PKA/PKC) in hyperexcitable neurons, favoring the stability of mood [13,14,15,16,17].

It has also been demonstrated that Li^+^ acts on the proliferation and differentiation of bone marrow mesenchymal stem cells, stimulating osteogenesis by activating different Wnt and Hedgehog (Hh) signaling pathways and inhibiting the enzyme glycogen synthase kinase-3β (GSK-3β) [18,19,20,21,22,23].

Recent experimental results have shown that Li^+^ stimulates the in vitro secretion of growth factors with proangiogenic activity and participates in angiogenesis in vivo [24]. It has also been shown that Li^+^ induces the proliferation, migration, and survival of endothelial cells by activating the Wnt/β-catenin canonical pathway [24,25,26,27]. These properties would constitute the rational base for the use of Li^+^ in biomaterials feasible for application in regenerative medicine and in the engineering of tissues with a high degree of vascularization such as bone tissue. However, the angiogenic effects of Li^+^ released from biomaterials have not yet been assessed. The studies that have examined the effect of the addition of Li^+^ into different biomaterials have focused on the evaluation of the physicochemical and structural properties as well as on the osteogenic potential of some of them [18,19,22,23,28,29,30,31,32,33,34,35,36,37,38,39,40,41]. In particular, the incorporation of Li^+^ into bioactive glasses was first described by Khorami et al., who partially substituted Na_2_O by variable amounts of Li_2_O (3, 7, and 12 wt %) in a bioactive glass in the SiO_2_-CaO-Na_2_O-P_2_O_5_ (45S5) system [30]. The results showed that although all the glasses developed were biocompatible with the cultured osteoblastic cells, the rate of formation of hydroxycarbonate apatite as an indicator of bioactivity was a dose-dependent phenomenon, which was delayed in the glasses with a higher content of Li_2_O [30]. These results are in agreement with the recent findings of Bruckner et al. using 45S5 microparticles (<38 µm) with 6.1 to 24.4% (mol %) of Li_2_O [33], as well as with the data reported by Miguez-Pacheco et al. for glass-ceramic scaffolds derived from the 45S5 bioactive glass with 2.5, 5, and 10 wt % of Li_2_O, which showed that a content of up to 5 wt % of Li_2_O keeps the bioactive behavior and thermal characteristics of the 45S5 glass [34]. On the other hand, it has been demonstrated that ion release from lithium-substituted BG can be tailored to induce an appropriate biological response [41]; for example, the partial replacement of Na_2_O by up to 5 wt % of Li_2_O in the 45S5 bioactive glass [34] ensures the release of Li^+^ within therapeutic levels for humans (0.5–1.2 mmol) [13,14]. 

In vitro assays are best suited to examine specific aspects of particular processes involved in angiogenesis such as the biochemical interactions that regulate endothelial cell proliferation, migration, or tubulogenesis [42]. Human umbilical vein endothelial cells (HUVECs) are in high demand for engineering blood vessels and vascularized tissues to treat diseases due to their low immunogenicity, and are an important cell model for studying endothelial cell biology [43]. Based on this background, the objective of this study was to assess the in vitro HUVECs response to ionic dissolution products of microparticles of 45S5 BG containing 5 wt % of Li_2_O (45S5.5Li). 

## 2. Materials and Methods

### 2.1. Development and Characterization of Bioactive Glass Microparticles

To prepare the glasses, we first selected a base composition that (a) was bioactive and (b) belonged to the SiO_2_-CaO-Na_2_O-P_2_O_5_ system. The mixture of raw materials was prepared in the necessary ratio to obtain the desired composition in a 45S5-type base glass containing (in wt %) 45% of SiO_2_, 24.5% Na_2_O, 24.5% CaO, and 6% P_2_O_5_, where Na_2_O was partially substituted by 5% Li_2_O (45S5.5Li). The mixtures were merged in a platinum crucible at 1500 °C using a Carbolite electric oven, keeping the same temperature for 3 h to allow the merger of the components and the homogenization of the glass. The melted glass was placed on graphite plates so as to induce fast cooling and thus prevent its crystallization. To verify the non-crystallization of the material, we carried out X-ray diffraction (XRD) using a Philips PW 1830 with Co tube, using Kα radiation of this element at 40 kV.

Considering the fast in vitro release of ions from the 45S5 bioactive glass when using particles smaller than or equal to 5 µm [10], in the present study, the glass blocks obtained were ground and reduced to powders of micrometric particles (<5 µm) and were then examined by scanning electronic microscopy (SEM, JEOL JSM 6480 LV) to evaluate their homogeneity and size.

### 2.2. Preparation of Culture Media Enriched with Ionic Dissolution Products from Bioactive Glasses

The M199 culture medium for endothelial cells (GIBCO, Carlsbad, CA, USA, Table S1) was used. The enriched culture media (M199 + 45S5 and M199 + 45S5.5Li) were prepared by incubating particles (<5 µm) from the bioactive glasses 45S5 or 45S5.5Li in M199 (1% w/v) for 72 h in an orbital shaker at 37 °C. The media were centrifuged and filtered with a 0.22 µm filter and then diluted by adding 50 µL of the enriched media M199 + 45S5 or M199 + 45S5.5Li in 100 µL of M199 medium. Their ionic composition was analyzed by ICP-MS.

### 2.3. Cell Culture

For the in vitro assays, HUVECs, kindly donated by Dr. M. Schattner (Academia Nacional de Medicina, Buenos Aires, Argentina), were used. HUVECs were grown in T75 flasks with M199 medium and 10% fetal bovine serum (FBS) and 50 µg mL^−1^ of gentamicin supplemented with 2 ng mL^−1^ of basic fibroblast growth factor (bFGF), and were kept in an incubator gassed with 5% CO_2_ in air at 37 °C. Then, the cells were washed with PBS and incubated at 37 °C for 5 min with 1 mL of a dilution of 0.017% w/v of trypsin and 0.18 mmol EDTA in PBS. Immediately after, 10 mL of M199 medium was added to dilute the trypsin and then centrifuged at 1000 rpm for 5 min. The supernatant was discarded and the cells were suspended in the appropriate volume of medium and counted in a Neubauer chamber to be used in the various assays detailed below:

*Proliferation Assay*: As reported in previous studies [10], HUVECs were sown in 96-well plates at a density of 5000 cells per well in 100 µL of M199 medium containing 10% of FBS and 50 µg mL^−1^ of gentamicin. At 24 h, 50 µL of M199 medium supplemented with the ionic dissolution products (M199 + 45S5; M199 + 45S5.5Li) or M199 medium enriched with 0.20 mmol of LiCl or NaCl (using the latter as control of the effect of osmolarity) was added in sextuplicate.

The proliferative response of HUVECs treated with the ionic dissolution products, LiCl and NaCl was also studied in the presence of quercetin (10 µM, dissolved in DMSO), an inhibitor of the Wnt/β-catenin canonical pathway.

As a positive control, we used M199 medium with bFGF, whereas for the negative control we used M199 medium without the ionic products or growth factors. At 24 h, 25 µL per well of the same medium, containing methyl-[3H]-thymidine at a final concentration of 2.5 µCi mL^−1^, was added and the incubation was continued for 24 additional hours. The assay was stopped by adding 50 µL of 6 M guanidinium chloride. Complete lysis of the cells was achieved through three cycles of freezing at −70 °C and thawing. Cellular DNA was collected in Whatman GFC filters with a Cell Harvester (Cell Harvester 8, Nunc, Rochester, NY, USA), fixed with 96% ethanol, and air-dried. The incorporated radioactivity was determined as described in Haro Durand et al. [10]. 

*Migration Assay*: To evaluate migration, we followed the method previously described [10]. HUVECs were seeded to confluence in 96-well culture plates overnight to ensure their adherence to the surface of the wells. Then, a gap was generated by means of a probe tip. After generating the gap, the cells were washed with PBS three times to remove detached cells and cell debris, and then were cultured for 8 h in M199 medium enriched with the ionic dissolution products (M199 + 45S5; M199 + 45S5.5Li) or 2 ng mL^−1^ bFGF (M199 + bFGF). As the negative control, we used M199 medium without the ionic dissolution products or bFGF. The effect of M199 medium enriched with 0.20 mmol LiCl or NaCl was also evaluated.

Photographic images were taken immediately after generating the gap (time zero) and after 8 h. Cell migration was quantified by means of an Image J image analyzer (NIH, Bethesda, MD, USA). The mean gap area was expressed as the recovery percentage (% R) of five wells, treated in the same way, using the following equation:

% *R* = [*1* − (*A_t_*/*A*_0_)] × *100* (1)

where *A*_0_ is the area of the gap at time 0 and *A_t_* is the area of the gap at 8 h.

*Transmigration Assay*: HUVECs were re-seeded in T75 flasks to obtain the desired number of cells for the experiment, and were then raised with EDTA and counted in a Neubauer chamber prior to centrifugation. At the beginning of the assay, 500 µL of M199 medium enriched with the ionic dissolution products of the bioactive glasses (M199 + 45S5; M199 + 45S5.5Li), M199 medium with 2 ng mL^−1^ bFGF (M199 + bFGF) as the positive control, and M199 medium without ionic dissolution products or bFGF as the negative control were placed, in triplicate, in each well of a transwell plate (Corning, New York, NY, USA). The effect of M199 medium enriched with 0.20 mmol of LiCl or NaCl was also studied. Then, the transwell inserts were placed and 20,000 cells were seeded in M199 medium (200 µL) in the upper compartment of each insert. After 8 h of incubation, the inserts were removed and placed in Crystal Violet for 10 min for fixation and staining of the cells. The inserts were then washed with water, and the residual cells which had not migrated through the microporous membrane (transmigration) were removed with a cotton swab. Cells were analyzed by means of an inverted optical microscope, and photos of two different areas were taken and the number of cells which had transmigrated to the lower side of the membranes was quantified.

*Tubulogenesis Assay in Matrigel™*: For the assay of tubulogenesis in Matrigel™, 60 µL of Matrigel™ (BD Biosciences, San Jose, CA, USA) was placed into each well of a 96-well plate and was left to solidify for 30 min at 37 °C before seeding the cells. HUVECs were trypsinized and raised, centrifuged, and counted in a Neubauer chamber. A total of 10,000 cells were seeded in each well onto the surface of the Matrigel™ in 150 µL of M199 medium supplemented with ionic dissolution products (M199 + 45S5; M199 + 45S5.5Li). The effect of M199 medium enriched with 0.20 mmol LiCl or NaCl was also evaluated. The assay was conducted in quadruplicate using M199 medium with bFGF as the positive control and M199 medium without ionic dissolution products or growth factor as the negative control.

The assay was monitored every hour for 8 h. At the end of the assay, the cells were evaluated by means of an inverted optical microscope and photographs of two fields per well were taken and the number of fully enclosed tubes present within each photographed area was quantified.

### 2.4. Enzyme Linked Immunosorbent Assay (ELISA)

An ELISA was conducted to measure the levels of secretion of proangiogenic cytokines, including bFGF, VEGF, IGF1, and TGF-β, among others. HUVECs were seeded to confluence in six-well culture plates. After reaching the cell monolayer, the cells were stimulated with M199 medium enriched with the ionic dissolution products (M199 + 45S5; M199 + 45S5.5Li), M199 medium with 2 ng mL^−1^ bFGF (M199 + bFGF) as the positive control, and M199 medium without ionic dissolution products or bFGF as the negative control. After 48 h, the supernatant was extracted and the assay continued according to the protocol of the commercial ELISA (EA-1011, Signosis, Holy Clear, CA, USA).

### 2.5. Determination of the Levels of β-Catenin by Western Blot

HUVECs were seeded to confluence in six-well culture plates. After reaching the monolayer, the cells were stimulated for 24 h with the ionic dissolution products of the bioactive glass 45S5.5Li (M199 + 45S5.5Li). The effect of the M199 medium enriched with 0.20 mmol of LiCl or NaCl was also studied. M199 medium without ionic dissolution products or bFGF was used as the negative control. At 24 h post-stimulation, the cells were washed with PBS three times to stop the reaction and were frozen at −20 °C. After 24 h, the cells were thawed and homogenized in 200 µL of RIPA buffer (50 mmol Tris-HCl, pH 7.4, 1% NP-40, 0.5% Na deoxycholate, 0.1% SDS, 150 mmol NaCl, 2 mmol EDTA, 2 mmol PMSF, and 50 mmol NaF), sonicated, and centrifuged for 15 min at 12,500 rpm. Then, 30 µg of total protein, determined by Bradford, was heated in seeding buffer (50 mmol Tris-HCl, pH 6.6; 2% SDS, 10% glycerol, and 0.05% bromophenol blue), for 5 min at 100 °C, and seeded in a 10% mini-polyacrylamide gel. Electrophoresis was performed as previously described [10]. The nitrocellulose membranes were blocked with 5% skim milk powder prepared in TBS (Tris 25 mmol, 150 mmol NaCl, 2 mmol KCl, pH 7.4), supplemented with 0.1% Tween-20 (TBS-T) for 1 h, washed with TBS-T three times, and incubated overnight with primary monoclonal anti-active β-catenin antibody, produced in mouse (1:500) (Millipore™ (Upstate™) clone: 8E7, 50-171-753, Waltham, MA, USA) or with a polyclonal anti-GAPDH IgG antibody produced in rabbit as a loading control (1:2500, Santa Cruz Biotechnology, sc-25778, Dallas, TX, USA). This incubation step was performed in TBS-T with 5% BSA at 4 °C. Then, the membranes were washed with TBS-T and detection was carried out by incubating for 1 h with the corresponding secondary antibodies: anti-mouse antibody produced in horse (1:2000, Vector PI-2000, Burlingame, CA, USA) and anti-rabbit antibody produced in goat (1:2500, Cell Signaling, #7074, Danvers, MA, USA) conjugated to peroxidase in TBS-T with 5% skim milk powder at room temperature. Subsequently, the membranes were washed and incubated for 1 min with a chemiluminescent peroxidase substrate solution (ECL™, Amersham Biosciences, GE Healthcare UK Ltd., Chalfont, Buckinghamshire, UK). The results were visualized by autoradiography of the chemiluminescent reaction.

### 2.6. Statistical Analysis

The results were statistically analyzed assuming α = 0.05 and β = 0.10 and the mean ± the standard deviation were calculated for all the data. The results were analyzed using analysis of variance (ANOVA) and Bonferroni as the post-hoc test. 

### 2.7. Ethical Principles

Institutional standards of biosafety and environmental care were followed. The collection of human umbilical venous endothelial cells (HUVECs) from umbilical cords was approved by the Institutional Review Board of the National Academy of Medicine, Buenos Aires, Argentina. 

## 3. Results and Discussion

### 3.1. Production and Characterization of Bioactive Glasses

The two compositions studied (45S5 and 45S5.5Li) were easily fused, producing homogeneous, colorless, and bubble-free glasses. Figure S1 shows X-ray diffractograms of the 45S5 and 45S5.5Li glasses. Both BGs showed the characteristic signal associated with the amorphous structure of a glass. The study by SEM showed a homogeneous distribution of microparticles <5 µm (Figure S2). The final composition of the as-prepared BGs (obtained by Inductively Coupled Plasma Mass Spectrometry chemical analysis) is given in Table S2. In the present study, the experimental compositions of the glass were close to the expected theoretical values, indicating that the glass network structure was formed as expected. The substitution of Na_2_O by Li_2_O was done on a wt % basis rather than the more appropriate mol % basis, to be able to compare the results with those of a previous study [34]. It has been shown, both theoretically and experimentally, that the partial substitution of Na_2_O for Li_2_O on a molar basis in a 45S5 glass favors the compaction of the glass matrix because the smaller ionic radius of Li^+^ (76 pm vs. 102 pm of Na^+^) and the greater tendency to form covalent Li-oxygen bonds determine the increase in oxygen density, defined as the concentration of oxygen atoms per volume unit of glass matrix [33,41,44]. The higher density of oxygen observed in glasses with Li_2_O content make the silicate network more compact, thus modifying the reactivity of the glass, reducing its solubility with the consequent reduction in the release of ions and delaying the formation of the apatite layer [33]. 

In a recent publication, Maçon et al [45]. have shown that when lithium citrate was used as the Li precursor, mesoporous glass containing Li as a network modifier was obtained, whereas the use of lithium nitrate produced a relatively dense glass-ceramic with the presence of lithium metasilicate, as shown by X-ray diffraction, magic angle spinning–solid state nuclear magnetic resonance (MAS NMR) spectroscopy, and nitrogen sorption data. Both glass and glass-ceramic released silica and Li^+^ ions in culture media, but the release rate was lower for the glass-ceramic. Both samples did not affect chondrocyte viability and proliferation [45].

### 3.2. Study of the Angiogenic Effects In Vitro 

#### 3.2.1. Culture Media Enriched with Ionic Dissolution Products from Bioactive Glasses 

Table 1 shows the ionic concentration of the culture media used in the different assays performed to evaluate the angiogenic effects in vitro. The concentration of Li^+^ released from 45S5.5Li microparticles is in agreement with the findings of Miguez-Pacheco et al. who used glass-ceramic scaffolds derived from the glass 45S5 with 2.5 and 5 wt % of Li_2_O incubated in SBF [34]. Similar results have been recently obtained in our laboratory when incubating glass-ceramic scaffolds derived from the glass 45S5.5Li in M199 medium. It is important to note that the concentration of Li^+^ released from the previously mentioned biomaterials is below the reported reference values at which cytotoxic effects are apparent (5–25 mmol) [18,24,46,47,48]. However, Bruckner et al. observed a higher release of Li^+^ (5.5 mmol) from microparticles (<38 µm or 300–500 µm) of the glass 45S5 with 12.2% in moles of Li_2_O incubated for 72 h in Tris buffer [33].

#### 3.2.2. Proliferation Assay

**Figure 1:**
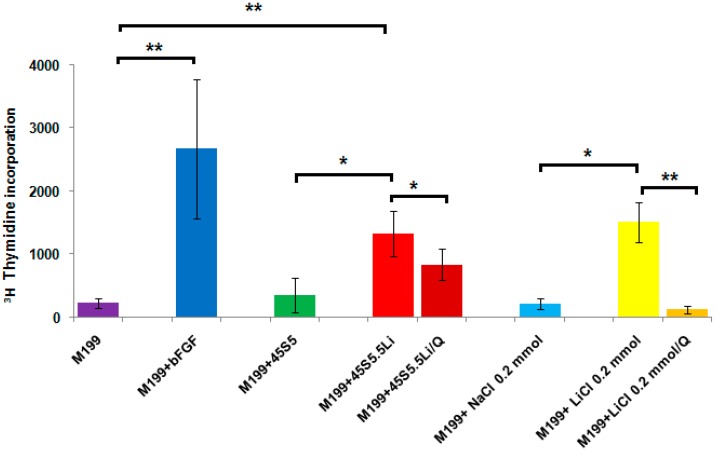
Proliferative response of human umbilical vein endothelial cells (HUVECs). Q: quercetin, an inhibitor of the Wnt/β-catenin canonical pathway. Data are means ± SD from one representative experiment of at least three independent experiments carried out in sextuplicate (* *p* < 0.05 and ** *p* < 0.01).

Figure 1 shows that the greatest proliferative response of human umbilical vein endothelial cells (HUVECs) was observed with the use of the M199 medium supplemented with bFGF (M199 + bFGF). In addition, as compared to the negative control (M199) and the M199 medium supplemented with the ionic dissolution products released from 45S5 (M199 + 45S5), at 48 h post-stimulation a statistically significant increase was observed in the proliferation of HUVECs treated with the ionic dissolution products from 45S5.5Li (M199 + 45S5.5Li).

To determine whether the presence of Li^+^ in the M199 medium enriched with the ionic dissolution products from 45S5.5Li (M199 + 45S5.5Li) could account for the effect observed in the proliferative capacity of HUVECs, we performed a proliferation assay with M199 medium enriched with 0.20 mmol of LiCl. The M199 medium supplemented with 0.20 mmol LiCl stimulated the in vitro proliferation of HUVECs, with no significant differences with the response observed with M199 + 45S5.5Li (Figure 1).

Literature data have shown that in human dermal microvascular endothelial cells (HDMEC), LiCl (0.20 or 1 mmol) significantly induced cell proliferation after incubation for 24 h [24]. Considering that it has also been shown that Li^+^ induces the proliferation and survival of endothelial cells through the activation of the Wnt/β-catenin canonical pathway [24], in the present study, we evaluated the proliferative response of HUVECs treated with M199 + 45S5.5Li or M199 + 0.20 mmol LiCl in the presence of an inhibitor of the Wnt/β-catenin canonical pathway (quercetin 10 µM dissolved in DMSO) [24]. The use of quercetin showed 92% of inhibition of the proliferation of HUVECs treated with 0.20 mmol LiCl (Figure 1b). However, the proliferative response of HUVECs treated with M199 + 45S5.5Li decreased only by 37% in the presence of the inhibitor (Figure 1).

These observations indicate that the greater proliferation of the endothelial cells evidenced when using the ionic dissolution products of the bioactive glass 45S5.5Li (M199 + 45S5.5Li) would be due not only to the presence of Li^+^ in the culture medium, but also to the release of other ions with proangiogenic capacity, in particular silicon (Si). Recent studies have shown that Si ions released from silica-based ceramic materials stimulate the secretion of pro-angiogenic growth factors, the proliferation of endothelial cells in vitro, and angiogenesis in vivo [6,49,50,51].

#### 3.2.3. Migration Assay

**Figure 2:**
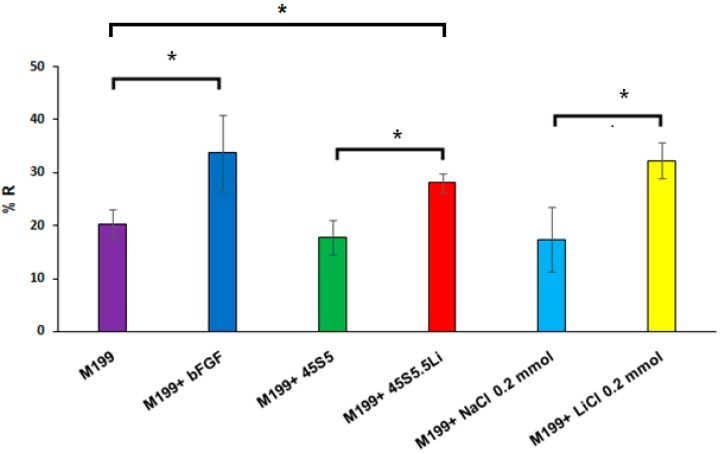
Recovery percentage (%R) of the gap by the migration of HUVECs. Data are means ± SD from one representative experiment of at least three independent experiments carried out in triplicate (* *p* < 0.05).

After stimulation with the ionic dissolution products of the bioactive glass 45S5.5Li (M199 + 45S5.5Li), the migratory response of HUVECs showed a statistically significant increase, managing to close the gap in approximately 28 ± 2% of the total area at 8 h post-stimulation, and this response was equivalent to using M199 medium supplemented with 0.20 mmol LiCl and M199 medium supplemented with bFGF (Figure 2 and Figure S3). Similar observations were made by Zeilbeck et al. [24] after the incubation of HDMEC with LiCl for 18 h. Quantification showed that following incubation with 0.20 or 1 mmol LiCl, the migration of HDMEC was significantly increased by 56% and 38%, respectively, compared with the untreated controls [24]. 

#### 3.2.4. Transmigration Assay

**Figure 3:**
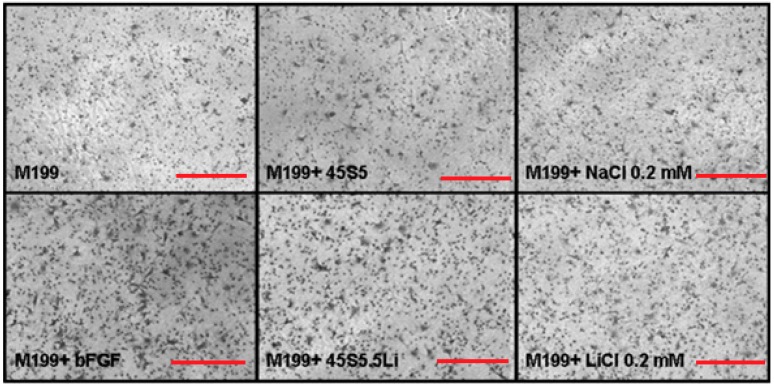
Photographs showing the cells that transmigrated in the Transwell^®^ system. Staining with Crystal Violet. Scale bar: 100 µm. The images are from one representative experiment of at least three independent experiments carried out in triplicate.

**Figure 4:**
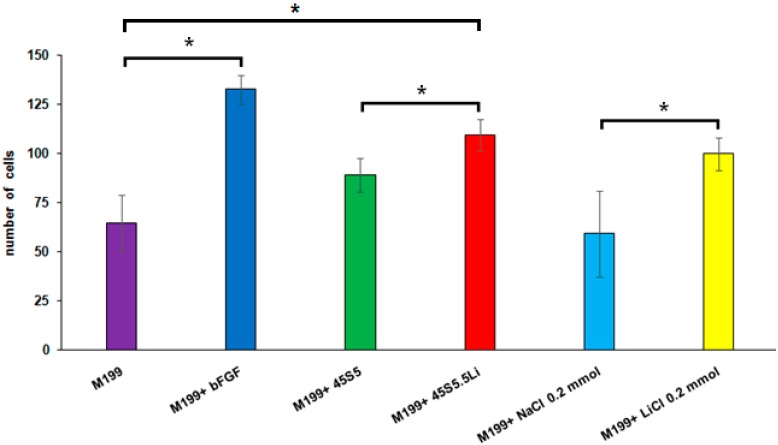
Number of HUVECs that transmigrated 8 h post-stimulation. Data are means ± SD from one representative experiment of at least three independent experiments carried out in triplicate (* *p* < 0.05).

In M199 medium supplemented with ionic dissolution products of 45S5.5Li (M199 + 45S5.5Li), the number of HUVECs that transmigrated after 8 h post-stimulation showed a statistically significant increase as compared to the negative control (M199) and M199 + 45S5, and this response was similar to that observed when using M199 + bFGF and M199 + 0.20 mmol LiCl (Figure 3 and Figure 4).

#### 3.2.5. Tubulogenesis Assay in Matrigel™

**Figure 5:**
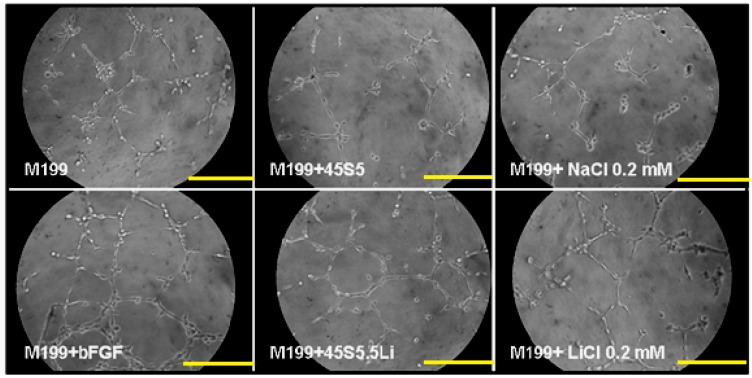
Microphotographs showing the formation of endothelial tubules in Matrigel™. Scale bar: 50 µm. The images are from one representative experiment of at least three independent experiments carried out in quadruplicate.

**Figure 6:**
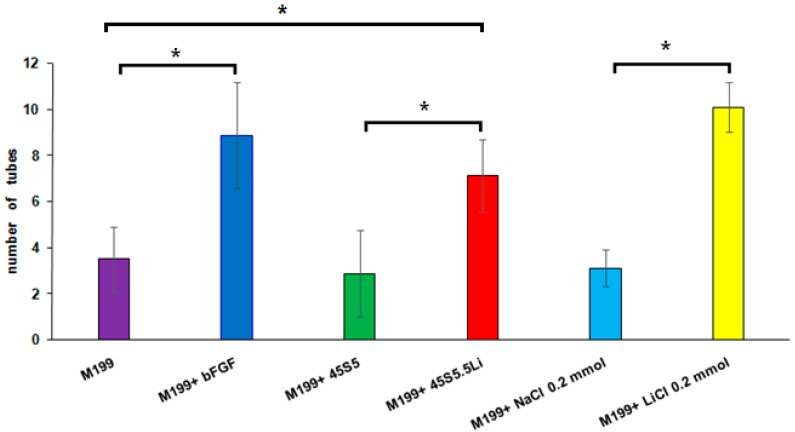
Number of endothelial tubules at 8 h post-stimulation. Data are means ± SD from one representative experiment of at least three independent experiments carried out in quadruplicate (* *p* < 0.05).

No evidence has at present arisen about the effects of Li^+^ in the Matrigel™ assay. At 8 h post-stimulation using M199 + 45S5.5Li, the capacity of HUVECs to form endothelial tubules in Matrigel™ was statistically significantly increased, and this was response similar to that achieved with the culture medium supplemented with bFGF and with that supplemented with 0.20 mmol LiCl (Figure 5 and Figure 6).

#### 3.2.6. Enzyme Linked Immunosorbent Assay (ELISA)

**Figure 7:**
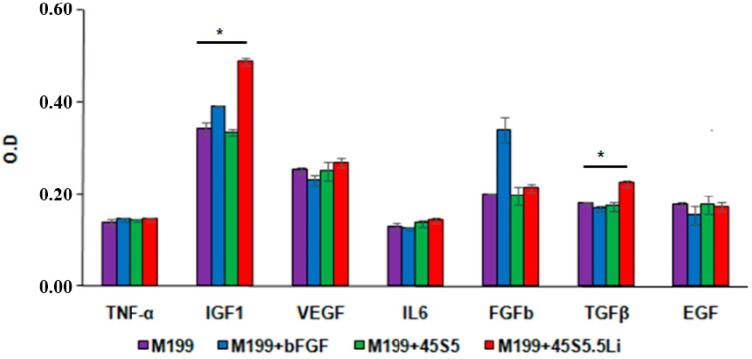
Levels of expression of proangiogenic cytokines. OD: optical density. Data are means ± SD from one representative experiment of at least three independent experiments carried out in triplicate (* *p* < 0.05).

With regards to the production of proangiogenic cytokines in HUVECs treated with the ionic dissolution products of the bioactive glass 45S5.5Li, the levels of expression of IGF1 (25%) and TGFβ (33%) were increased (Figure 7), whereas those of the other cytokines analyzed, such as VEGF, showed no increase. This response is in agreement with that described by Zeilbeck et al. and Guo et al*.* in endothelial cells treated with 0.20 mmol LiCl [24,25]. However, Guo et al. [25] described an enhanced expression of VEGF in brain endothelial cells only after incubation with very high concentrations of LiCl (10 or more mmol). 

#### 3.2.7. Determination of the Levels of β-Catenin by Western Blot

**Figure 8:**
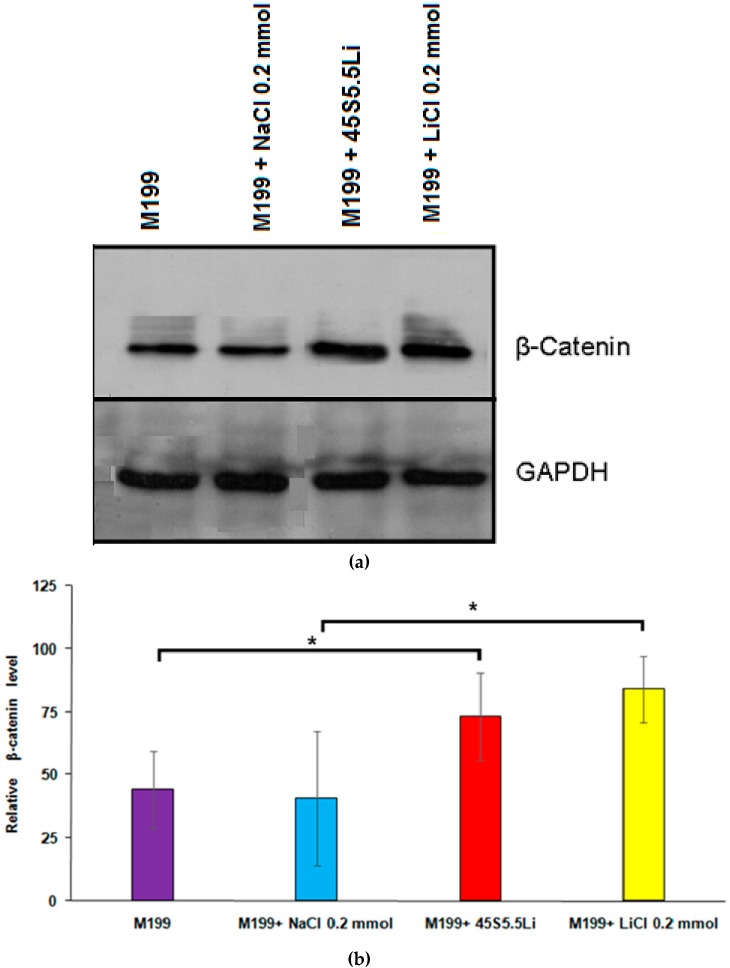
Western blot (**a**) and relative expression of β-catenin (**b**) in HUVECs. Data are means ± SD from one representative experiment of at least three independent experiments carried out in triplicate (* *p* < 0.05).

At 24 h post-stimulation, the expression of β-catenin in HUVECs treated with M199 + 45S5.5Li showed a statistically significant increase in comparison to the control, and this response was equivalent to that obtained with the use of M199 +0.20 mmol LiCl (Figure 8). In line with our results, Zeilbeck et al. [24] described that the incubation of human dermal microvascular endothelial cells with 0.20 mmol LiCl caused an increase in the levels of β-catenin and their translocation to nuclei, indicating an activation of the Wnt/β-catenin signaling pathway. 

### 3.3. Summary

The results showed that HUVECs treated with ion dissolution products of BG microparticles (<5 µm) of the composition 45S5.5Li exhibit a proliferative and migratory response and have the capacity to form tubules in vitro. Therefore, the ionic dissolution products released from 45S5.5Li could act as inorganic angiogenic agents, thus becoming a promising alternative to costly and potentially dangerous growth factors [52,53].

Considering the results obtained in the present study and the previously published findings [54,55,56,57,58,59], the stimulation of the angiogenic response would be due to the synergistic effect that determines the release of several ions from microparticles of the bioactive glass 45S5.5Li on the production of proangiogenic cytokines (IGF1 and TGF-β) and the activation of the Wnt/β-catenin canonical pathway. It has been previously described that the proangiogenic effects of TGF-β are associated with the expression of ALK1 receptors and to the activation of signaling cascades that trigger the proliferation and migration of endothelial cells via the phosphorylation of Smad 1/5 [60,61], and that IGF1 acts through the IGF1 receptor by activating the migration and tubulogenesis of endothelial cells through the Ras/Raf/ERK pathway [62]. In addition, IGF1 is involved in the activation of β-catenin [54,55,56], generating a positive feedback circuit that contributes to the proliferation, migration, and survival of endothelial cells [24,25,26,27]. On the other hand, it is important to point out that Si and Li^+^ ions also promote osteogenesis and bone repair [18,19,50,63,64,65,66,67,68,69]. It would be therefore of interest to further evaluate the angiogenic and osteogenic effects of 45S5.5Li particles of different sizes (nano- and micrometric) and/or scaffolds derived from the bioactive glass 45S5.5Li by using more complex cell cultures such as the co-culture of osteoblasts with endothelial cells, as well as in experimental models of bone repair in vivo.

Our findings demonstrate for the first time that HUVECs have greater migratory and proliferative response and capacity to form tubules in vitro after stimulation with the ionic dissolution products released from the bioactive glass 45S5.5Li. In addition, we observed the activation of the Wnt/β-catenin canonical pathway and an increase in the levels of expression of proangiogenic cytokines (IGF1 and TGF-β). The results of the present work thus demonstrated that the ionic dissolution products released from the bioactive glass 45S5.5Li stimulate HUVECs behavior in vitro. Such bioactive glass is thus attractive as an inorganic angiogenic agent for applications in different strategies of regenerative medicine and for the engineering of tissues that require a high degree of vascularization.

## Figures and Tables

**Table 1:** Elemental concentration determined by inductively coupled plasma mass spectrometry (ICP-MS) (mean ± SD) ^a)^.

Group	Li (mmol)	Si (mmol)	P (mmol)	Ca (mmol)	Na (mmol)
M199	Bld ^b)^	0.85 ± 0.03	6.81 ± 0.13	0.21 ± 0.05	191 ± 3
M199 + 45S5	Bld	1.53 ± 0.07	16.30 ± 0.35	0.65 ± 0.02	291 ± 4
M199 + 45S5.5Li	0.20 ± 0.01	1.46 ± 0.07	18.10 ± 0.39	0.72 ± 0.02	276 ± 4

^a)^ SD: standard deviation; ^b)^ Bld: below the limit of detection.

## Supplementary Materials

The following are available online at www.mdpi.com/1996-1944/10/7/740/s1. Figure S1: X-ray diffraction (XRD) study of the bioactive glasses, Figure S2: Scanning electron microscopy (SEM) of 45S5.5Li microparticles, Figure S3: Migration assay, Table S1: Formulation detail of medium M199, Table S2: Chemical composition of the as-prepared bioactive glasses.
