# Supplementary material for: In Vitro Human Umbilical Vein Endothelial Cells Response to Ionic Dissolution Products from Lithium-Containing 45S5 Bioactive Glass
Source: Materials (Basel). 2017 Jul 3;10(7):740. doi: 10.3390/ma10070740 (PMC5551783; doi:10.3390/ma10070740)
Supplement: Supplementary file 1 [file materials-10-00740-s001.pdf]

Supplementary Materials: In vitro Human Umbilical Vein Endothelial Cells Response to Ionic Dissolution Products from Lithium-containing 45S5 Bioactive Glass

Luis A. Haro Durand, Gabriela E. Vargas, Rosa Vera-Mesones, Alberto Baldi, María P. Zago, María A. Fanovich, Aldo R. Boccaccini and Alejandro Gorustovich *

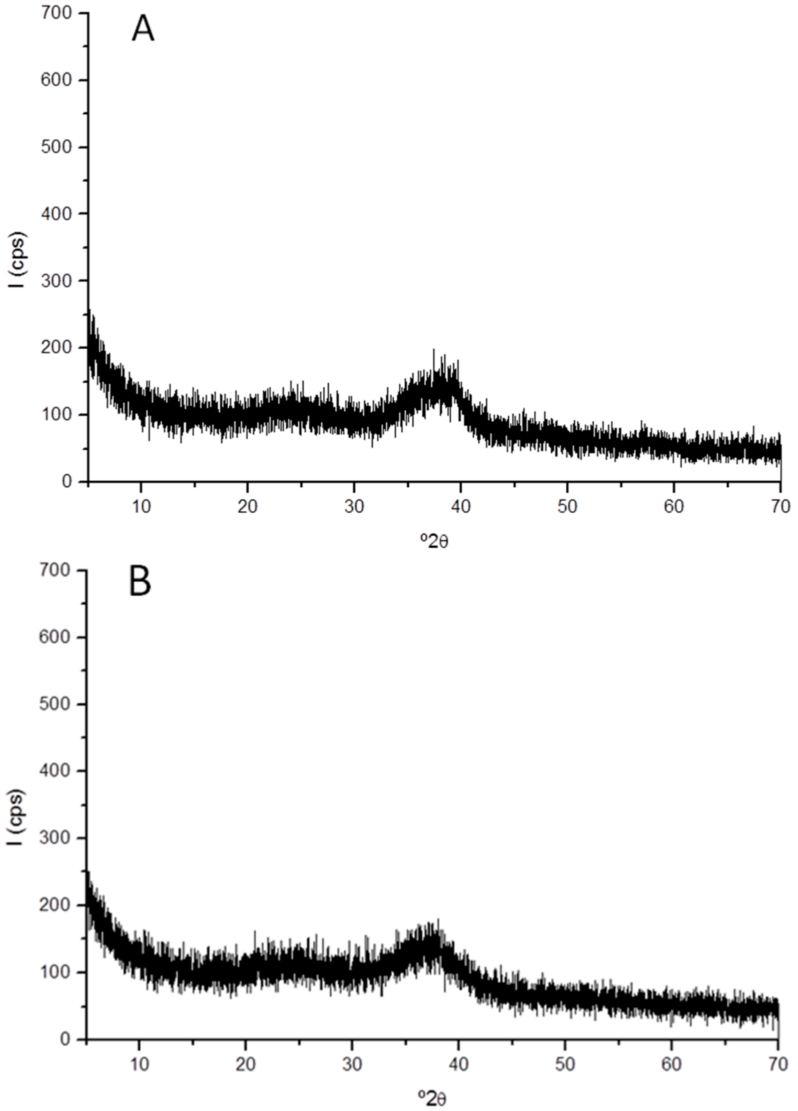

Figure S1. X-ray diffraction (XRD) study of the bioactive glasses 45S5 (A) and 45S5.5Li (B) showing the absence of crystalline phases.

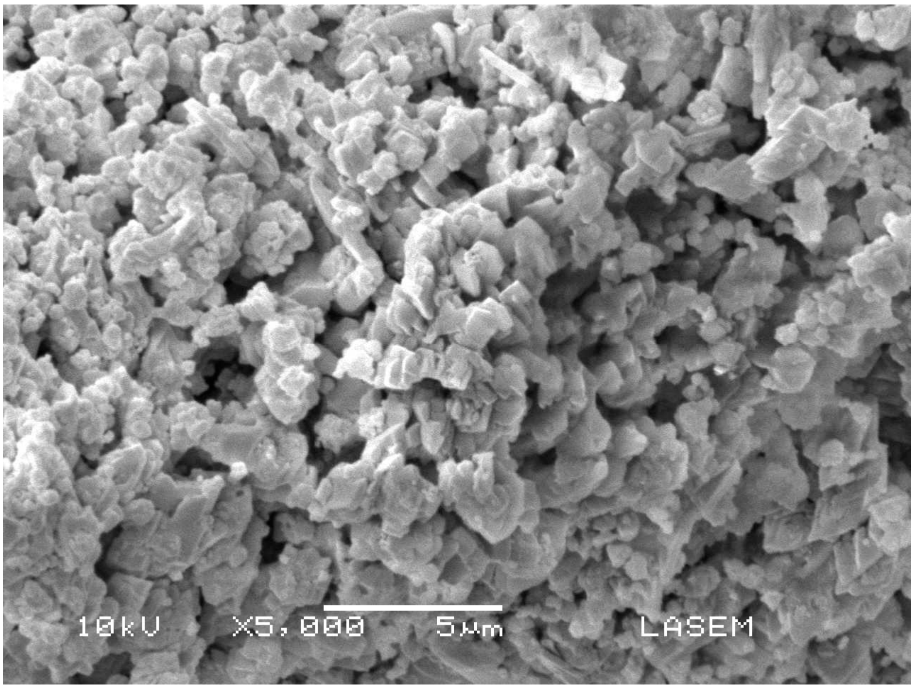

Figure S2. Scanning electron microscopy (SEM) of 45S5.5Li microparticles.

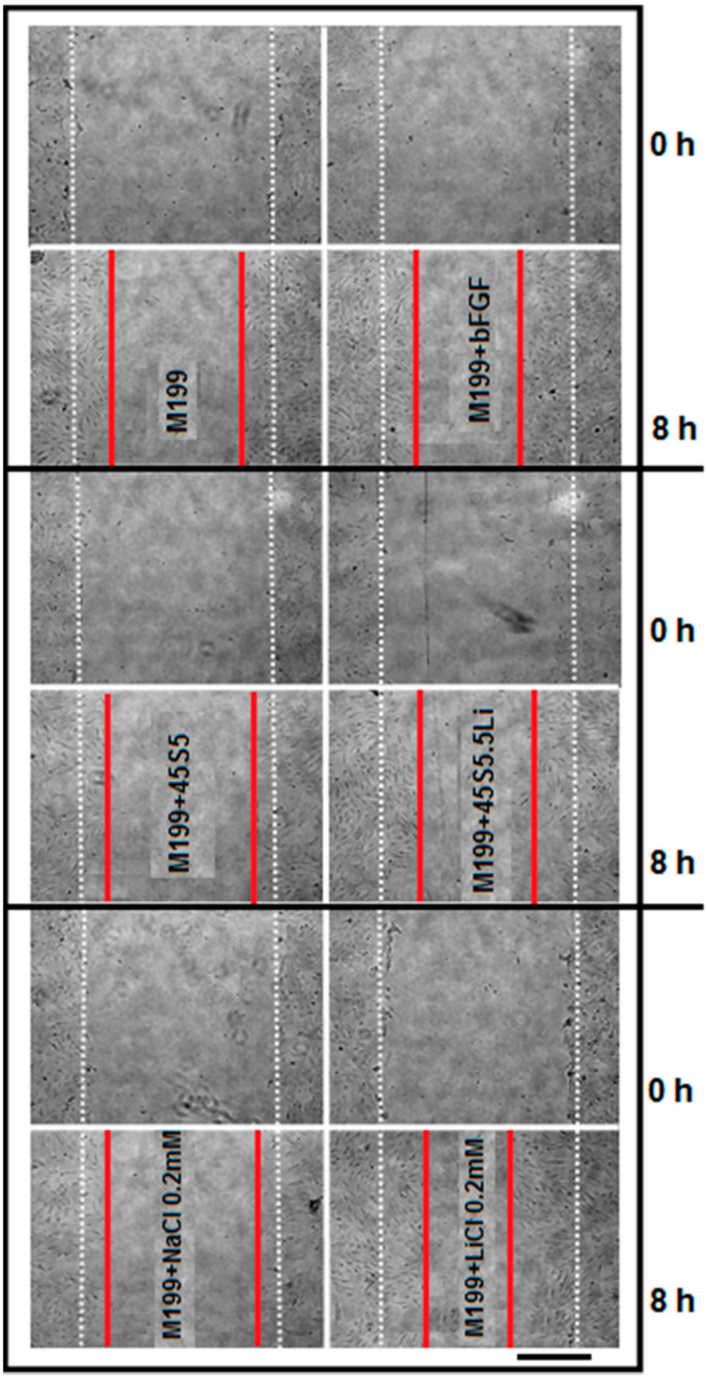

Figure S3. Migratory response of HUVECs. Dotted lines indicate the gap at t_0, while the red full lines delimit the coating of the gap by migration of HUVECs. Scale bar:100µm.

Table S1. Formulation detail of medium M199.

COMPONENTS	Molecular Weight	Concentration (mg/L)	mM
**Amino Acids**			
Glycine	75	50	0.667
L-Alanine	89	25	0.281
L-Arginine hydrochloride	211	70	0.332

L-Aspartic acid	133	30	0.226
L-Cysteine hydrochloride-H2O	176	0.1	0.000568
L-Cystine 2HCl	240	26	0.108
L-Glutamic Acid	147	75	0.51
L-Glutamine	146	100	0.685
L-Histidine hydrochloride-H2O	210	21.88	0.104
L-Hydroxyproline	131	10	0.0763
L-Isoleucine	131	40	0.305
L-Leucine	131	60	0.458
L-Lysine hydrochloride	183	70	0.383
L-Methionine	149	15	0.101
L-Phenylalanine	165	25	0.152
L-Proline	115	40	0.348
L-Serine	105	25	0.238
L-Threonine	119	30	0.252
L-Tryptophan	204	10	0.049
L-Tyrosine disodium salt dihydrate	261	58	0.222
L-Valine	117	25	0.214
**Vitamins**			
Alpha-tocopherol Phosphate	702	0.01	0.0000142
Ascorbic Acid	176	0.05	0.000284
Biotin	244	0.01	0.000041
Choline chloride	140	0.5	0.00357
D-Calcium pantothenate	477	0.01	0.000021
Folic Acid	441	0.01	0.0000227
Menadione (Vitamin K3)	172	0.01	0.0000581
Niacinamide	122	0.025	0.000205
Nicotinic acid (Niacin)	123	0.025	0.000203
Para-Aminobenzoic Acid	137	0.05	0.000365
Pyridoxal hydrochloride	204	0.025	0.000123
Pyridoxine hydrochloride	206	0.025	0.000121

Riboflavin	376	0.01	0.0000266
Thiamine hydrochloride	337	0.01	0.0000297
Vitamin A (acetate)	328	0.1	0.000305
Vitamin D2 (Calciferol)	397	0.1	0.000252
i-Inositol	180	0.05	0.000278
Inorganic Salts			
Calcium Chloride (CaCl2) (anhyd.)	111	200	1.8
Ferric nitrate (Fe(NO3)-9H2O)	404	0.7	0.00173
Magnesium Sulfate (MgSO4) (anhyd.)	120	97.67	0.814
Potassium Chloride (KCl)	75	400	5.33
Sodium Chloride (NaCl)	58	6800	117.24
Sodium Phosphate monobasic (NaH2PO4-H2O)	138	140	1.01
Other Components			
Adenine sulfate	404	10	0.0248
Adenosine 5'-phosphate	347	0.2	0.000576
Adenosine 5'-triphosphate	605	1	0.00165
Cholesterol	387	0.2	0.000517
D-Glucose (Dextrose)	180	1000	5.56
Deoxyribose	134	0.5	0.00373
Glutathione (reduced)	307	0.05	0.000163
Guanine hydrochloride	188	0.3	0.0016
Hypoxanthine Na	136	0.4	0.00294
Phenol Red	376.4	20	0.0531
Ribose	150	0.5	0.00333
Sodium Acetate	82	50	0.61
Thymine	126	0.3	0.00238
Tween 80®		20	∞
Uracil	112	0.3	0.00268
Xanthine-Na	152	0.3	0.00197

Table S2. Chemical composition of the as-prepared bioactive glasses determined by

inductively coupled plasma mass spectrometry (ICP-MS) (in wt %).

	**Li_2O**	**SiO_2**	**P_2O_5**	**CaO**	**Na_2O**
**45S5**	-	44.97	5.96	24.53	24.55
**45S5.5Li**	4.95	44.95	5.99	24.55	19.55

© 2017 by the authors. Submitted for possible open access publication under the terms and conditions of the Creative Commons Attribution (CC BY) license (http://creativecommons.org/licenses/by/4.0/).
